# (μ-Piperazine-1,4-dicarbodithioato-κ^4^ *S*,*S*′:*S*′′,*S*′′′)bis­[triphenyl­tin(IV)] dichloro­methane solvate

**DOI:** 10.1107/S1600536808025890

**Published:** 2008-08-16

**Authors:** Pavel Poplaukhin, Edward R. T. Tiekink

**Affiliations:** a Department of Chemistry, University of Texas at San Antonio, One UTSA Circle, San Antonio, TX 78249-0698, USA

## Abstract

The dinuclear centrosymmetric title compound, [Sn_2_(C_6_H_5_)_6_(C_6_H_8_N_2_S_4_)]·CH_2_Cl_2_, features a distorted *cis*-trigonal–bipyramidal coordination geometry for Sn based on a C_3_S_2_ donor set. The dinuclear mol­ecule lies across a centre of inversion. The solvent dichloro­methane mol­ecule is disordered about a centre of inversion.

## Related literature

For a review of tin dithio­carbamates, see: Tiekink (2008 ▶). For a related structure, see: Yin *et al.* (2002 ▶). For analysis of trigonal–bipyramidal geometries, see: Addison *et al.* (1984 ▶).
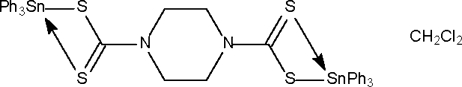


## Experimental

### 

#### Crystal data

[Sn_2_(C_6_H_5_)_6_(C_6_H_8_N_2_S_4_)]·CH_2_Cl_2_
*M* *_r_* = 1021.37
Monoclinic, 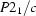

*a* = 14.681 (5) Å
*b* = 10.758 (3) Å
*c* = 13.470 (4) Å
β = 90.379 (6)°
*V* = 2127.3 (11) Å^3^
*Z* = 2
Mo *K*α radiation
μ = 1.53 mm^−1^
*T* = 98 (2) K
0.35 × 0.15 × 0.01 mm

#### Data collection

Rigaku AFC12κ/SATURN724 diffractometer
Absorption correction: multi-scan (*ABSCOR*; Higashi, 1995 ▶) *T*_min_ = 0.354, *T*_max_ = 1 (expected range = 0.346–0.977)
14400 measured reflections
4389 independent reflections
4021 reflections with *I* > 2σ(*I*)
*R*_int_ = 0.048

#### Refinement

*R*[*F* ^2^ > 2σ(*F* ^2^)] = 0.048
*wR*(*F* ^2^) = 0.113
*S* = 1.10
4389 reflections
244 parameters
H-atom parameters constrained
Δρ_max_ = 0.77 e Å^−3^
Δρ_min_ = −1.12 e Å^−3^

### 

Data collection: *CrystalClear* (Rigaku/MSC, 2005 ▶); cell refinement: *CrystalClear*; data reduction: *CrystalClear*; program(s) used to solve structure: *SHELXS97* (Sheldrick, 2008 ▶); program(s) used to refine structure: *SHELXL97* (Sheldrick, 2008 ▶); molecular graphics: *ORTEPII* (Johnson, 1976 ▶); software used to prepare material for publication: *SHELXL97*.

## Supplementary Material

Crystal structure: contains datablocks global, I. DOI: 10.1107/S1600536808025890/ng2481sup1.cif

Structure factors: contains datablocks I. DOI: 10.1107/S1600536808025890/ng2481Isup2.hkl

Additional supplementary materials:  crystallographic information; 3D view; checkCIF report

## Figures and Tables

**Fig. 1.**
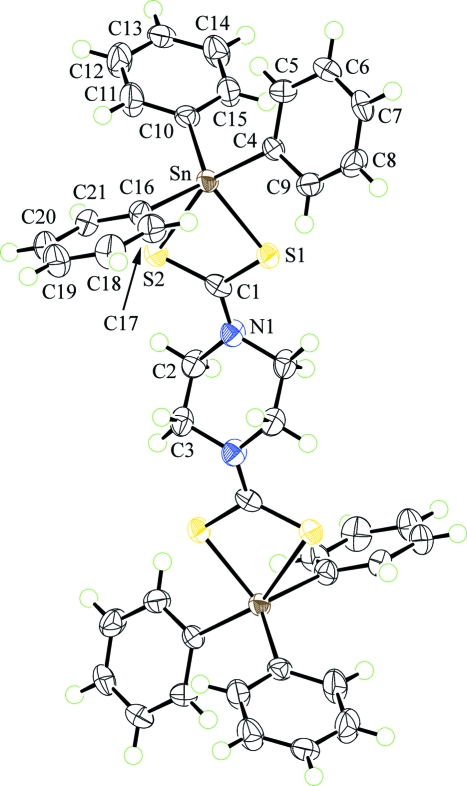
Molecular structure of (I) showing the crystallographic numbering scheme. Displacement ellipsoids are shown at the 70% probability level. Unlabelled atoms are related by the symmetry operation i: -x, 1-y, 1-z. The disordered dichloromethane molecule is omitted.

## supplementary crystallographic information

### Comment

Tin dithiocarbamates continue to attract interest owing to their variety of applications (Tiekink, 2008). The title compound, Ph_3_SnS_2_CN(CH_2_CH_2_)_2_NCS_2_SnPh_3_, has been reported previously as a methanol solvate (Yin *et al.*, 2002). The present structure (I) has been isolated as a dichloromethane solvate, Fig. 1. The molecule is centrosymmetric so that the Ph_3_Sn entities lie to either side of the pyrrolidine ring which adopts a chair conformation. The dithiocarbamate ligand coordinates in an asymmetric mode, forming Sn—S1 and Sn—S2 distances of 2.4699 (13) and 3.0715 (13) Å, respectively. The coordination geometry is based on a distorted trigonal bipyramid as indicated by the value of τ = 0.64 (Addison *et al.*, 1984).

### Experimental

The title compound was prepared by following a literature procedure (Yin *et al.*, 2002). Colourless crystals were isolated by the slow evaporation of a dichloromethane solution of (I); m.p. 487–489 K (crystal turned opaque at 363–368 K). TGA: two steps, First mass loss 7.2% (onset 388.3 K, midpoint 392.6 K, endset 396.9 K) corresponds to loss CH_2_Cl_2_ (8.2% theoretical). Second mass loss 69.3% (onset 558.5 K, midpoint 620 K, endset 680 K), corresponds to decomposition to SnS (total experimental mass loss 76.5% cf. theoretical value 70.5%). IR (cm^-1^): 1427, 1416 (strong, C═N), 1214 (strong, C—S).

### Refinement

The H atoms were geometrically placed (C—H = 0.95–0.99 Å) and refined as riding with *U*_iso_(H) = 1.2*U*_eq_(C). The solvent dichloromethane molecule was disordered about a centre of inversion and was modelled with anisotropic displacement parameters.

### Crystal data

**Table:**

[Sn_2_(C_6_H_5_)_6_(C_6_H_8_N_2_S_4_)]·CH_2_Cl_2_	*F*_000_ = 1020
*M**_r_* = 1021.37	*D*_x_ = 1.594 Mg m^−^^3^
Monoclinic, *P*2_1_/*c*	Mo *K*α radiation λ = 0.71069 Å
Hall symbol: -P 2ybc	Cell parameters from 13756 reflections
*a* = 14.681 (5) Å	θ = 2.4–40.7º
*b* = 10.758 (3) Å	µ = 1.53 mm^−^^1^
*c* = 13.470 (4) Å	*T* = 98 (2) K
β = 90.379 (6)º	Plate, colourless
*V* = 2127.3 (11) Å^3^	0.35 × 0.15 × 0.02 mm
*Z* = 2	

### Data collection

**Table:**

Rigaku AFC12κ/SATURN724 diffractometer	4389 independent reflections
Radiation source: fine-focus sealed tube	4021 reflections with *I* > 2σ(*I*)
Monochromator: graphite	*R*_int_ = 0.048
*T* = 98(2) K	θ_max_ = 26.5º
ω scans	θ_min_ = 2.4º
Absorption correction: multi-scan(ABSCOR; Higashi, 1995)	*h* = −17→18
*T*_min_ = 0.354, *T*_max_ = 1	*k* = −13→13
14400 measured reflections	*l* = −16→16

### Refinement

**Table:**

Refinement on *F*^2^	Secondary atom site location: difference Fourier map
Least-squares matrix: full	Hydrogen site location: inferred from neighbouring sites
*R*[*F*^2^ > 2σ(*F*^2^)] = 0.048	H-atom parameters constrained
*wR*(*F*^2^) = 0.113	*w* = 1/[σ^2^(*F*_o_^2^) + (0.04*P*)^2^ + 8.4194*P*] where *P* = (*F*_o_^2^ + 2*F*_c_^2^)/3
*S* = 1.10	(Δ/σ)_max_ = 0.001
4389 reflections	Δρ_max_ = 0.77 e Å^−^^3^
244 parameters	Δρ_min_ = −1.12 e Å^−^^3^
Primary atom site location: structure-invariant direct methods	Extinction correction: none

### Special details

**Table:**

Geometry. All esds (except the esd in the dihedral angle between two l.s. planes) are estimated using the full covariance matrix. The cell esds are taken into account individually in the estimation of esds in distances, angles and torsion angles; correlations between esds in cell parameters are only used when they are defined by crystal symmetry. An approximate (isotropic) treatment of cell esds is used for estimating esds involving l.s. planes.
Refinement. Refinement of F^2^ against ALL reflections. The weighted R-factor wR and goodness of fit S are based on F^2^, conventional R-factors R are based on F, with F set to zero for negative F^2^. The threshold expression of F^2^ > 2sigma(F^2^) is used only for calculating R-factors(gt) etc. and is not relevant to the choice of reflections for refinement. R-factors based on F^2^ are statistically about twice as large as those based on F, and R- factors based on ALL data will be even larger.

### Fractional atomic coordinates and isotropic or equivalent isotropic displacement parameters (Å^2^)

**Table:**

	*x*	*y*	*z*	*U*_iso_*/*U*_eq_	Occ. (<1)
Sn	0.23132 (2)	0.37950 (3)	0.17164 (2)	0.02056 (11)	
S1	0.10733 (8)	0.32408 (10)	0.28651 (9)	0.0237 (3)	
S2	0.12861 (8)	0.59723 (11)	0.26696 (9)	0.0244 (3)	
N1	0.0190 (3)	0.4872 (4)	0.3970 (3)	0.0250 (9)	
C1	0.0795 (3)	0.4757 (4)	0.3239 (3)	0.0228 (9)	
C2	−0.0077 (3)	0.6087 (4)	0.4381 (4)	0.0264 (11)	
H2A	−0.0745	0.6193	0.4320	0.032*	
H2B	0.0220	0.6761	0.4002	0.032*	
C3	0.0208 (3)	0.6161 (4)	0.5471 (4)	0.0260 (10)	
H3A	0.0880	0.6128	0.5525	0.031*	
H3B	0.0001	0.6961	0.5756	0.031*	
C4	0.2559 (3)	0.1881 (4)	0.1326 (3)	0.0212 (9)	
C5	0.2678 (3)	0.1546 (5)	0.0335 (4)	0.0238 (10)	
H5	0.2690	0.2174	−0.0160	0.029*	
C6	0.2780 (3)	0.0316 (5)	0.0060 (4)	0.0284 (11)	
H6	0.2846	0.0103	−0.0620	0.034*	
C7	0.2785 (3)	−0.0610 (4)	0.0782 (4)	0.0284 (11)	
H7	0.2869	−0.1453	0.0596	0.034*	
C8	0.2666 (4)	−0.0303 (5)	0.1768 (4)	0.0320 (12)	
H8	0.2664	−0.0936	0.2259	0.038*	
C9	0.2549 (3)	0.0934 (4)	0.2042 (4)	0.0270 (10)	
H9	0.2462	0.1139	0.2721	0.032*	
C10	0.1869 (3)	0.4710 (4)	0.0402 (3)	0.0231 (10)	
C11	0.2262 (4)	0.5821 (5)	0.0088 (4)	0.0413 (14)	
H11	0.2767	0.6157	0.0442	0.050*	
C12	0.1920 (4)	0.6440 (5)	−0.0742 (5)	0.0424 (15)	
H12	0.2184	0.7205	−0.0944	0.051*	
C13	0.1199 (4)	0.5948 (4)	−0.1273 (4)	0.0272 (10)	
H13	0.0966	0.6377	−0.1836	0.033*	
C14	0.0818 (4)	0.4841 (5)	−0.0989 (4)	0.0344 (12)	
H14	0.0324	0.4497	−0.1356	0.041*	
C15	0.1166 (4)	0.4223 (5)	−0.0151 (4)	0.0307 (11)	
H15	0.0908	0.3449	0.0038	0.037*	
C16	0.3474 (3)	0.4550 (4)	0.2475 (4)	0.0238 (10)	
C17	0.4094 (3)	0.3737 (5)	0.2921 (4)	0.0297 (11)	
H17	0.4011	0.2865	0.2860	0.036*	
C18	0.4830 (4)	0.4199 (5)	0.3453 (4)	0.0369 (12)	
H18	0.5247	0.3640	0.3759	0.044*	
C19	0.4966 (4)	0.5477 (5)	0.3543 (4)	0.0378 (13)	
H19	0.5475	0.5791	0.3904	0.045*	
C20	0.4352 (3)	0.6280 (5)	0.3103 (4)	0.0308 (11)	
H20	0.4439	0.7151	0.3165	0.037*	
C21	0.3608 (3)	0.5833 (4)	0.2570 (4)	0.0247 (10)	
H21	0.3191	0.6397	0.2270	0.030*	
C22	0.5409 (12)	0.0134 (14)	0.5721 (11)	0.066 (4)	0.50
H22A	0.6046	0.0434	0.5764	0.080*	0.50
H22B	0.5172	0.0006	0.6400	0.080*	0.50
Cl1	0.4667 (2)	0.1259 (2)	0.4994 (2)	0.0958 (9)	

### Atomic displacement parameters (Å^2^)

**Table:**

	*U*^11^	*U*^22^	*U*^33^	*U*^12^	*U*^13^	*U*^23^
Sn	0.02652 (19)	0.01628 (17)	0.01887 (18)	−0.00089 (12)	−0.00114 (13)	0.00000 (11)
S1	0.0281 (6)	0.0167 (5)	0.0263 (6)	−0.0002 (4)	0.0029 (5)	−0.0026 (4)
S2	0.0294 (6)	0.0191 (5)	0.0247 (6)	0.0000 (5)	0.0020 (5)	−0.0025 (4)
N1	0.027 (2)	0.0180 (19)	0.030 (2)	0.0038 (16)	0.0035 (17)	0.0006 (16)
C1	0.024 (2)	0.023 (2)	0.021 (2)	0.0046 (18)	−0.0024 (18)	−0.0055 (18)
C2	0.025 (2)	0.019 (2)	0.035 (3)	0.0045 (18)	0.010 (2)	−0.0009 (19)
C3	0.024 (2)	0.018 (2)	0.036 (3)	0.0017 (18)	0.007 (2)	−0.001 (2)
C4	0.026 (2)	0.015 (2)	0.023 (2)	0.0014 (17)	0.0030 (18)	−0.0032 (17)
C5	0.024 (2)	0.027 (2)	0.021 (2)	0.0037 (19)	0.0010 (18)	−0.0005 (19)
C6	0.032 (3)	0.030 (3)	0.023 (3)	0.001 (2)	−0.001 (2)	−0.008 (2)
C7	0.029 (3)	0.018 (2)	0.038 (3)	−0.0012 (19)	−0.002 (2)	−0.007 (2)
C8	0.043 (3)	0.021 (2)	0.032 (3)	0.001 (2)	0.007 (2)	0.006 (2)
C9	0.035 (3)	0.024 (2)	0.022 (2)	0.001 (2)	0.003 (2)	0.0007 (19)
C10	0.032 (2)	0.018 (2)	0.019 (2)	0.0025 (18)	0.0003 (19)	−0.0009 (17)
C11	0.057 (4)	0.033 (3)	0.034 (3)	−0.019 (3)	−0.024 (3)	0.012 (2)
C12	0.060 (4)	0.029 (3)	0.039 (3)	−0.014 (3)	−0.013 (3)	0.013 (2)
C13	0.041 (3)	0.022 (2)	0.019 (2)	0.006 (2)	−0.001 (2)	−0.0006 (19)
C14	0.039 (3)	0.029 (3)	0.035 (3)	−0.004 (2)	−0.016 (2)	0.002 (2)
C15	0.038 (3)	0.024 (2)	0.030 (3)	−0.007 (2)	−0.009 (2)	0.003 (2)
C16	0.018 (2)	0.027 (2)	0.026 (3)	−0.0031 (18)	−0.0007 (18)	−0.0023 (19)
C17	0.028 (3)	0.031 (3)	0.030 (3)	0.001 (2)	−0.004 (2)	0.001 (2)
C18	0.029 (3)	0.036 (3)	0.045 (3)	0.002 (2)	−0.006 (2)	0.004 (3)
C19	0.032 (3)	0.040 (3)	0.041 (3)	−0.011 (2)	−0.005 (2)	−0.005 (3)
C20	0.026 (3)	0.032 (3)	0.034 (3)	−0.009 (2)	−0.002 (2)	−0.003 (2)
C21	0.023 (2)	0.023 (2)	0.027 (3)	−0.0027 (19)	0.0017 (19)	0.0021 (19)
C22	0.102 (12)	0.050 (8)	0.048 (9)	0.001 (8)	0.023 (8)	0.001 (7)
Cl1	0.155 (3)	0.0477 (11)	0.0854 (17)	0.0173 (13)	0.0274 (17)	0.0024 (11)

### Geometric parameters (Å, °)

**Table:**

Sn—C10	2.125 (5)	C10—C15	1.373 (7)
Sn—C16	2.141 (5)	C10—C11	1.394 (7)
Sn—C4	2.157 (4)	C11—C12	1.392 (8)
Sn—S1	2.4699 (13)	C11—H11	0.9500
Sn—S2	3.0715 (13)	C12—C13	1.378 (8)
S1—C1	1.756 (5)	C12—H12	0.9500
S2—C1	1.680 (5)	C13—C14	1.370 (7)
N1—C1	1.337 (6)	C13—H13	0.9500
N1—C3^i^	1.466 (6)	C14—C15	1.403 (7)
N1—C2	1.473 (6)	C14—H14	0.9500
C2—C3	1.527 (7)	C15—H15	0.9500
C2—H2A	0.9900	C16—C17	1.396 (7)
C2—H2B	0.9900	C16—C21	1.400 (7)
C3—N1^i^	1.466 (6)	C17—C18	1.385 (7)
C3—H3A	0.9900	C17—H17	0.9500
C3—H3B	0.9900	C18—C19	1.395 (8)
C4—C5	1.394 (6)	C18—H18	0.9500
C4—C9	1.403 (7)	C19—C20	1.380 (8)
C5—C6	1.382 (7)	C19—H19	0.9500
C5—H5	0.9500	C20—C21	1.389 (7)
C6—C7	1.392 (7)	C20—H20	0.9500
C6—H6	0.9500	C21—H21	0.9500
C7—C8	1.380 (7)	C22—Cl1^ii^	1.784 (15)
C7—H7	0.9500	C22—Cl1	1.896 (16)
C8—C9	1.392 (7)	C22—H22A	0.9900
C8—H8	0.9500	C22—H22B	0.9900
C9—H9	0.9500	Cl1—C22^ii^	1.784 (15)
			
C10—Sn—C16	117.43 (18)	C8—C9—H9	119.7
C10—Sn—C4	106.86 (18)	C4—C9—H9	119.7
C16—Sn—C4	110.16 (18)	C15—C10—C11	118.3 (5)
C10—Sn—S1	114.23 (13)	C15—C10—Sn	120.1 (4)
C16—Sn—S1	112.36 (13)	C11—C10—Sn	121.7 (4)
C4—Sn—S1	92.74 (12)	C12—C11—C10	120.4 (5)
C10—Sn—S2	81.15 (12)	C12—C11—H11	119.8
C16—Sn—S2	84.41 (13)	C10—C11—H11	119.8
C4—Sn—S2	156.03 (12)	C13—C12—C11	120.3 (5)
S1—Sn—S2	63.66 (4)	C13—C12—H12	119.9
C1—S1—Sn	97.41 (16)	C11—C12—H12	119.9
C1—S2—Sn	79.09 (16)	C14—C13—C12	120.2 (5)
C1—N1—C3^i^	125.3 (4)	C14—C13—H13	119.9
C1—N1—C2	122.6 (4)	C12—C13—H13	119.9
C3^i^—N1—C2	111.8 (4)	C13—C14—C15	119.3 (5)
N1—C1—S2	123.6 (4)	C13—C14—H14	120.4
N1—C1—S1	117.0 (4)	C15—C14—H14	120.4
S2—C1—S1	119.4 (3)	C10—C15—C14	121.6 (5)
N1—C2—C3	109.6 (4)	C10—C15—H15	119.2
N1—C2—H2A	109.7	C14—C15—H15	119.2
C3—C2—H2A	109.7	C17—C16—C21	119.2 (5)
N1—C2—H2B	109.7	C17—C16—Sn	118.9 (4)
C3—C2—H2B	109.7	C21—C16—Sn	121.9 (4)
H2A—C2—H2B	108.2	C18—C17—C16	120.2 (5)
N1^i^—C3—C2	110.3 (4)	C18—C17—H17	119.9
N1^i^—C3—H3A	109.6	C16—C17—H17	119.9
C2—C3—H3A	109.6	C17—C18—C19	120.6 (5)
N1^i^—C3—H3B	109.6	C17—C18—H18	119.7
C2—C3—H3B	109.6	C19—C18—H18	119.7
H3A—C3—H3B	108.1	C20—C19—C18	119.1 (5)
C5—C4—C9	118.2 (4)	C20—C19—H19	120.4
C5—C4—Sn	120.2 (3)	C18—C19—H19	120.4
C9—C4—Sn	121.5 (3)	C19—C20—C21	121.0 (5)
C6—C5—C4	121.2 (5)	C19—C20—H20	119.5
C6—C5—H5	119.4	C21—C20—H20	119.5
C4—C5—H5	119.4	C20—C21—C16	119.8 (5)
C5—C6—C7	119.8 (5)	C20—C21—H21	120.1
C5—C6—H6	120.1	C16—C21—H21	120.1
C7—C6—H6	120.1	Cl1^ii^—C22—Cl1	102.9 (8)
C8—C7—C6	120.1 (5)	Cl1^ii^—C22—H22A	111.2
C8—C7—H7	120.0	Cl1—C22—H22A	111.2
C6—C7—H7	120.0	Cl1^ii^—C22—H22B	111.2
C7—C8—C9	120.0 (5)	Cl1—C22—H22B	111.2
C7—C8—H8	120.0	H22A—C22—H22B	109.1
C9—C8—H8	120.0	C22^ii^—Cl1—C22	77.1 (8)
C8—C9—C4	120.6 (5)		
			
C10—Sn—S1—C1	−69.6 (2)	Sn—C4—C9—C8	176.9 (4)
C16—Sn—S1—C1	67.4 (2)	C16—Sn—C10—C15	175.8 (4)
C4—Sn—S1—C1	−179.5 (2)	C4—Sn—C10—C15	51.5 (4)
S2—Sn—S1—C1	−3.88 (16)	S1—Sn—C10—C15	−49.5 (4)
C10—Sn—S2—C1	126.8 (2)	S2—Sn—C10—C15	−105.3 (4)
C16—Sn—S2—C1	−114.3 (2)	C16—Sn—C10—C11	−5.6 (5)
C4—Sn—S2—C1	15.0 (4)	C4—Sn—C10—C11	−129.8 (5)
S1—Sn—S2—C1	4.10 (17)	S1—Sn—C10—C11	129.2 (4)
C3^i^—N1—C1—S2	−175.8 (4)	S2—Sn—C10—C11	73.4 (4)
C2—N1—C1—S2	−2.8 (7)	C15—C10—C11—C12	2.7 (9)
C3^i^—N1—C1—S1	4.6 (7)	Sn—C10—C11—C12	−176.0 (5)
C2—N1—C1—S1	177.6 (4)	C10—C11—C12—C13	−1.2 (10)
Sn—S2—C1—N1	174.5 (4)	C11—C12—C13—C14	−0.4 (9)
Sn—S2—C1—S1	−5.9 (2)	C12—C13—C14—C15	0.5 (8)
Sn—S1—C1—N1	−173.0 (3)	C11—C10—C15—C14	−2.6 (8)
Sn—S1—C1—S2	7.3 (3)	Sn—C10—C15—C14	176.1 (4)
C1—N1—C2—C3	−116.5 (5)	C13—C14—C15—C10	1.1 (8)
C3^i^—N1—C2—C3	57.4 (5)	C10—Sn—C16—C17	−143.2 (4)
N1—C2—C3—N1^i^	−56.4 (5)	C4—Sn—C16—C17	−20.6 (4)
C10—Sn—C4—C5	18.7 (4)	S1—Sn—C16—C17	81.2 (4)
C16—Sn—C4—C5	−110.0 (4)	S2—Sn—C16—C17	139.8 (4)
S1—Sn—C4—C5	135.0 (4)	C10—Sn—C16—C21	39.4 (5)
S2—Sn—C4—C5	125.3 (3)	C4—Sn—C16—C21	162.0 (4)
C10—Sn—C4—C9	−157.4 (4)	S1—Sn—C16—C21	−96.1 (4)
C16—Sn—C4—C9	74.0 (4)	S2—Sn—C16—C21	−37.6 (4)
S1—Sn—C4—C9	−41.1 (4)	C21—C16—C17—C18	0.2 (8)
S2—Sn—C4—C9	−50.8 (6)	Sn—C16—C17—C18	−177.2 (4)
C9—C4—C5—C6	0.3 (7)	C16—C17—C18—C19	−0.5 (9)
Sn—C4—C5—C6	−175.9 (4)	C17—C18—C19—C20	0.6 (9)
C4—C5—C6—C7	−1.4 (7)	C18—C19—C20—C21	−0.4 (9)
C5—C6—C7—C8	1.6 (8)	C19—C20—C21—C16	0.0 (8)
C6—C7—C8—C9	−0.5 (8)	C17—C16—C21—C20	0.0 (7)
C7—C8—C9—C4	−0.6 (8)	Sn—C16—C21—C20	177.4 (4)
C5—C4—C9—C8	0.7 (7)	Cl1^ii^—C22—Cl1—C22^ii^	0.000 (2)

Symmetry codes: (i) −*x*, −*y*+1, −*z*+1; (ii) −*x*+1, −*y*, −*z*+1.
